# Skin Blood Perfusion and Oxygenation Colour Affect Perceived Human Health

**DOI:** 10.1371/journal.pone.0005083

**Published:** 2009-04-01

**Authors:** Ian D. Stephen, Vinet Coetzee, Miriam Law Smith, David I. Perrett

**Affiliations:** Perception Lab, School of Psychology, University of St Andrews, Scotland, United Kingdom; London School of Economics, United Kingdom

## Abstract

Skin blood perfusion and oxygenation depends upon cardiovascular, hormonal and circulatory health in humans and provides socio-sexual signals of underlying physiology, dominance and reproductive status in some primates. We allowed participants to manipulate colour calibrated facial photographs along empirically-measured oxygenated and deoxygenated blood colour axes both separately and simultaneously, to optimise healthy appearance. Participants increased skin blood colour, particularly oxygenated, above basal levels to optimise healthy appearance. We show, therefore, that skin blood perfusion and oxygenation influence perceived health in a way that may be important to mate choice.

## Introduction

Body or ornament colour reflects underlying physiology in several species. Moreover, these colour cues are perceived by conspecifics and used as social and sexual signals.

Red colouration, based on haemoglobin, is thought to provide signals of physiological state in birds. Blood perfusion causes red colouration in the mouths of canary nestlings. The gape becomes redder as the level of food deprivation increases. Nestlings with brighter red mouths are provided with more food by the parents, suggesting that the red colouration is acting as a signal between offspring and parents [1]. It has been noted that red, haemoglobin-based skin flushing is present in at least 29 bird genera, and may signal health or hormonal condition or social status [2].

Several species of non-human primates exhibit skin reddening that reflects aspects of physiological health, hormonal and reproductive status. There is also evidence that conspecifics react to these skin colour cues. Male rhesus macaques show facial reddening in the mating season, in response to increased levels of testosterone [3]. Similarly, in some macaque species the anogenital skin of females reddens in response to increased levels of ovarian oestrogen. This sexual skin becomes reddest in the periovulatory (most fertile) period (reviewed in [4]). Increased skin redness of male faces and female anogenital regions of macaques attracts greater visual attention from opposite sex individuals, suggesting that redness acts as a signal of condition and reproductive status [4], [5].

In male mandrills, elevated testosterone associated with increased social dominance rank leads to enhanced facial redness [6]. Other males avoid violent conflict with individuals with brighter red faces than themselves, suggesting that this increased redness is a signal of social status between dominant and subordinate males [7]. Female facial redness in mandrills is associated with fertility across the oestrus cycle and reproductive quality [8].

In humans too, skin redness caused by skin vasodilation and vascularisation has connections to physiological status including health. Additionally, blood oxygenation state is related to health status and affects skin colour. In women, increased sex hormone levels are associated with increased skin vascularisation [9] and vasodilatory response [10], which arterializes the blood in the skin [11]. The cutaneous vasodilator system becomes more responsive with physical training [12], but is impaired in type 2 diabetes [13] and hypertension [14]. Increased blood oxygenation is associated with increased aerobic fitness [15] whereas increased blood deoxygenation is associated with hypoxia and can lead to cyanosis (blue tinted skin), which is indicative of coronary and respiratory illness [16].

In humans, there is evidence that colouration is interpreted by observers as a cue to underlying physiological health or quality. The distribution of pigment colour (blood and melanin) in the skin can affect the apparent health, age and attractiveness of human faces [17], [18]. The relative lightness of the features and the facial skin affects the attractiveness and apparent femininity/masculinity of faces [19]. Participants who wear red are seen as more likely to win sporting contests [20] and experience more success in sporting contests [21], [22], possibly because redness may be interpreted as a cue to dominance [23] or anger in humans [24]. Women who wear red are seen as more attractive by men [25]. Previous work has not, however, addressed the impact of overall pigment colouration on the apparent health of human faces.

A decrease in blood perfusion below normal levels (pallor) is associated with ill health (especially anaemia; [26] which may also indicate other illnesses such as malaria [27]). Whether raising skin blood perfusion above normal levels has a beneficial or detrimental effect on perceived health is unclear. Moreover, it is not clear whether the appearance of health is affected by the subtle colour changes associated with blood oxygenation state. These questions are addressed here. We hypothesise that increased blood colour in human facial skin will be perceived as healthy, and that oxygenated blood colour will appear healthier than deoxygenated blood colour.

Changes in blood perfusion may be more difficult to detect in dark skinned ethnic groups [28]. Skin darkness has been shown to affect both social perceptions [29] and attractiveness ratings [30] of faces. It may be expected therefore that facial reddening is perceived differently in faces of differing ethnicity, and by light and dark skinned observers. A cross-cultural study is performed to examine this hypothesis.

## Materials and Methods

This work was approved by the University of St Andrews Ethics Committee. All participants gave prior, informed consent in writing.

**Figure 1:**
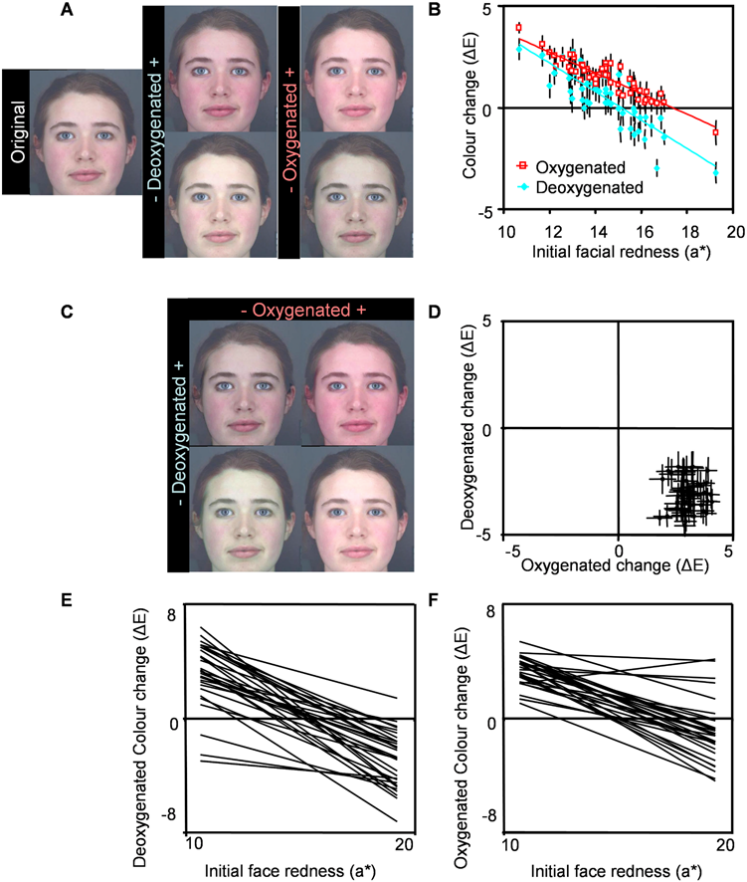
Effect of skin redness and blood colour on apparent health of faces. (A) Untransformed image and images pair showing low (−) and high (+) endpoints of oxygenated and deoxygenated blood colour transforms. (B) Participants increase deoxygenated and oxygenated blood colour to optimise healthy appearance. Initial facial redness (a*) correlates with oxygenated (r = −0.911; p<0.001; R^2^ = 0.83) and deoxygenated (r = −0.831; p<0.001; R^2^ = 0.69) blood colour change applied to optimize health appearance. (C) Endpoints of the two-dimensional oxygenated versus deoxygenated blood colour transform. (D) Two-dimensional colour transform applied to optimize healthy appearance (ΔE mean±SE). (E) Regression lines relating initial face redness to deoxygenated colour change applied to optimise healthy appearance for each participant. (F) Regression lines relating initial face redness to oxygenated colour change applied to optimise healthy appearance for each participant.

To test predictions, skin portions of colour-calibrated [31], Caucasian facial images were transformed [32] along empirically measured pigment colour axes: oxygenated and deoxygenated blood (Fig. 1A). Caucasian participants were presented with facial images on calibrated CRT monitors, and asked to manipulate their colour to “make the face as healthy as possible”. These trials were initially performed along one colour axis at a time. Further participants were tested with two-dimensional control of face colour, allowing simultaneous manipulation of oxygenated and deoxygenated blood colour axes (Fig. 1C).

A cross-cultural study was performed to investigate whether the ethnicity of faces and participants affects the preference for redness in human faces. Faces of various ethnicities were transformed along the CIELab a* (redness) colour axis. Caucasian UK-based and black South African participants adjusted the red colour of the faces under the same instructions as above.

### (a) Photography

51 Caucasian participants (21 male, 30 female; aged 18–22) were photographed, without skin makeup and with neutral expressions, in a booth painted Munsell N5 grey, illuminated with three Verivide F20 T12/D65 daylight simulation bulbs in high-frequency fixtures (Verivide, UK), to reduce the effects of flicker. The booth was located in a room with no other lighting. A Munsell N5 painted board was placed over the shoulders and a GretagMacbeth Mini ColorChecker colour chart was included in the frame. Images were colour corrected using a least-squares transform, from an 11-expression polynomial expansion [31] of camera RGB values for 24 ColorChecker patches to the manufacturer-specified CIELab values of the same patches. This achieved a mean colour error (ΔE) of 2.44 between the 24 manufacturer stated colour values and the colour values obtained from the corrected images. (ΔE is the Euclidean distance between two colour points in CIELab space, and is the standard method of quoting colour differences in CIELab colour space.)

For the cross-cultural study, photographs were taken of undergraduate students (10 white male, 10 white female, 7 East Asian male, 9 East Asian female, 6 black female, 5 South Asian female, 3 mixed ethnicity female), using a Fujifilm FinePix S2Pro digital SLR camera, fitted with a Nikon 60 mm fixed length lens, and a Nikon Canfield lens-mounted flash. Participants were seated, facing the camera, in front of a grey screen, and asked to maintain a neutral expression. Additional images were taken with a GretagMacbeth ColorChecker chart in the frame. Colour measurements (in the CIELab colour space) were taken of the colour patches in the image and directly with a Konica Minolta CM2600d spectrophotometer. Linear transforms were used to colour calibrate the images (achieving a post-calibration mean colour error [ΔE] of 6.704).

Matlab was used to calculate mean CIELab values across skin pixels for each face image (defining initial CIELab face colour).

### (b) Pigment colour measurement

Empirical measurements of the impact of oxygenated and deoxygenated blood on skin colour were made.

#### Deoxygenated blood

10 further participants (5 male, 5 female; aged 24–35) were recruited. A spectrophotometer (Konika Minolta CM2600d) reading (CIELab colour space, d65 illuminant, 10° illumination angle, SCI) was taken of the first dorsal interosseous region of the left hand (chosen for relative lack of visible tendons and veins), after they had stood with hands by their sides for 30 s (hyperaemia – high blood content), and again after holding their hands above their heads for 10 s (hypoaemia – low blood content). The elevated blood content of the lowered arm is due largely to deoxygenated blood [33].

#### Oxygenated blood

10 further participants (2 male, 8 female; aged 19–25) were recruited. Spectrophotometer measurements were made of the first dorsal interosseous region of the left hand, after 2 minutes sitting with hands resting on a desk (hypoaemia), and again after five minutes of sitting with their left hand in hot water (45–50°C, causing a high degree of hyperaemia with arterial blood [34]; the measured skin region was gently and quickly dried before measurement). The colour changes induced by increased perfusion with oxygenated and deoxygenated blood is similar between the hand and the face.

### (c) Image manipulation

Face-shaped masks were produced in Matlab, one representing each of the high and low pigment colours (see Table S1). Each face image (n = 51) was manipulated by the difference in colour between each of the pairs of masks [32]. Thirteen images were generated in equal steps from high to low colour, for each of the pigments. For 2D transforms, each of the thirteen images was transformed along the second colour dimension, giving 169 images per face (Fig. 1E). Hair, eyes, clothing and background remained constant.

**Figure 2:**
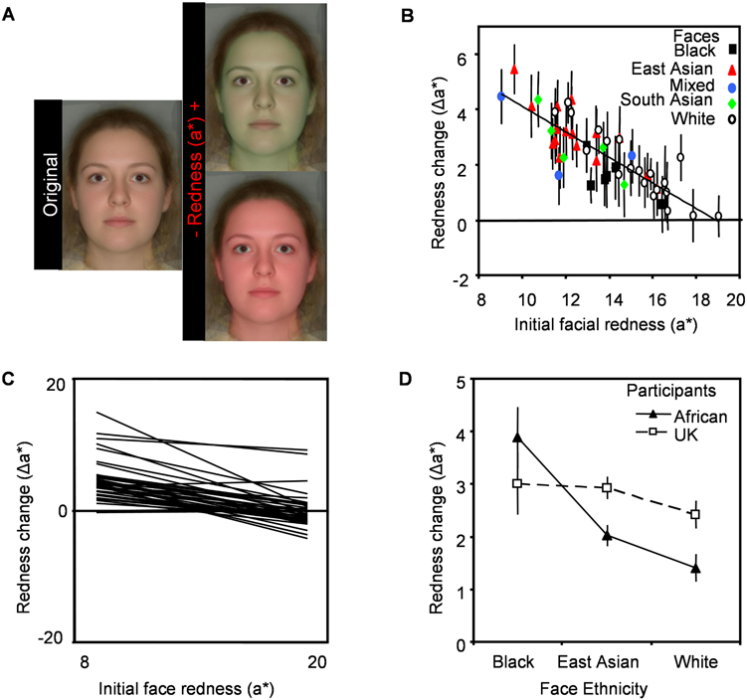
Effect of skin colour on apparent health of faces of various ethnicity. (A) Untransformed image and image pair showing endpoints of skin redness transform (±16 units on the CIELab a* axis). Images shown are composites of many faces. Stimuli used were photographs of real individuals. (B) Participants increase redness to optimise healthy appearance of faces. Initial facial redness correlates with redness change applied to optimize healthy appearance (r = −0.832; p<0.001; R^2^ = 0.69). Different coloured symbols represent different ethnic groups of faces. (C) Regression lines relating initial face redness to redness change applied to optimise healthy appearance for each individual participant. (D) African participants increase redness in black faces more than other faces. Both African and UK-based participants increase redness in all three ethnic group faces to optimise healthy appearance.

In the cross-cultural studies, faces were transformed along the CIELab a* (redness) colour axis, producing thirteen images in equal steps from −16 to +16 units of a* from the original face colour (fig. 2A).

### (d) Experimentation

Participants were Caucasian for manipulations along single blood colour axes: (14 male, 16 female, aged 18–24) and for manipulations on two blood colour axes: (15 male, 14 female, aged 18–25).

Participants were presented with the stimuli, one face at a time, in random order on a CRT monitor (colour calibrated using a ColorVision Spyder 2Pro to mean ΔE of 2.32 for a range of skin tones reflecting faces of various ethnicity). A computer program allowed participants to manipulate the colour of the facial skin along a single pigment colour axis to achieve optimum healthy appearance. Each participant saw each of the 51 faces once for the oxygenated and once for the deoxygenated blood colour transforms. Two-dimensional trials allowed participants to manipulate colour in two dimensions simultaneously using horizontal and vertical mouse movements. Each participant saw each of the 51 faces once in the two dimensional trials.

For the cross-cultural study, single-axis CIELab a* transformation trials were presented to black South African (10 male, 10 female; aged 19–29) and Caucasian UK-based (7 male, 11 female; aged 18–34) participants.

### (e) Statistical Methods

Statistical analyses were performed using SPSS version 16. Mean colour changes applied to each face were calculated (by face dataset). Mean colour changes applied by each participant were also calculated (by participant dataset). One-sample t-tests (H_0_: no colour change) were used to evaluate the overall colour changes. General Linear Mixed Models were used to test for the effect of face sex, participant sex and the interaction between the two on colour change applied (dependent variable = colour change; fixed factors = face sex, participant sex; random factor = participant ID; covariates = initial face colour: L*, a* and b*). All main effects were included in the model. Participant ID was nested within participant sex. The interaction between participant sex and face sex was included in the model. General Linear Mixed Modelling was used to test for relationships between initial face colour and colour change applied to optimise healthy appearance (dependent variable = colour change; random factor = participant ID; covariates = initial face colour: L*, a* and b*). The model was defined to include all main effects, as well as the interactions between participant ID and each colour covariate. In the cross-cultural study, General Linear Mixed Modelling was used to test for effects of face ethnicity, participant ethnicity and the interaction between the two on colour change applied (dependent variable = redness change applied; fixed factor = face ethnicity; random factors = participant ethnicity, participant ID; covariates = initial face colour: L*, a* and b*). The model included all main effects. Participant ID was nested within participant ethnicity. The interaction between ethnicity of image and ethnicity of face was included in the model.

## Results

With single axis transforms participants could simulate an increase or decrease in skin perfusion with one type of blood. With these transforms, participants increased oxygenated (Fig. 1B; ΔE = 1.52±0.13; t_50_ = 12.101, p<0.001) and deoxygenated blood colour (Fig. 1B; ΔE = 0.51±0.19; t_50_ = 2.702, p = 0.009) to optimize healthy appearance (see also Table S4). The initial redness of the face (CIELab a*) explained the colour change participants applied to optimize healthy appearance, with initially less red faces increased in blood colour more than initially redder faces. Initial facial redness affected the amount of deoxygenated (F_1,1410_ = 279.782; p<0.001; Fig. 1E) and oxygenated (F_1,1410_ = 237.100; p<0.001; Fig. 1F) blood colour change applied to optimise healthy appearance (Table S5). Interactions between initial face redness and participant ID were found for oxygenated (F_29,1410_ = 2.057; p = 0.001) and deoxygenated (F_29,1410_ = 1.579; p = 0.026) blood colour trials, suggesting that different participants are influenced by the initial redness of the face to different extents. To examine this effect more closely, individual Pearson's correlations were performed for each participant, examining the relationship between colour change and initial face redness. The magnitude of the correlations varied across participants but all participants in the deoxygenated trials (100%; Fig. 1E) and all but one (97%; the exception did not show a significant relationship) in the oxygenated trials (Fig. 1F) showed negative relationships between these variables. Thus blood colour is enhanced most in faces starting out low in redness (i.e. appearing low in skin blood perfusion).

Effects of face sex were found, with more oxygenated blood colour added to female faces than to male faces (F_1,1495_ = 4.095; p<0.043) and more deoxygenated blood colour added to male faces than to female faces (F_1,1495_ = 4.870; p = 0.027), possibly suggesting that female faces are more sensitive to blood oxygenation colour. No effect of participant sex or interaction between face sex and participant sex was found (Table S2). Oxygenated and deoxygenated blood colour was added to both male and female faces and by both male and female participants to optimise healthy appearance.

Oxygenated blood colour was more beneficial to apparent health than deoxygenated blood colour. All but one of the faces (98%) appeared healthier when oxygenated blood colour was elevated, whereas 66% appeared healthier with elevated deoxygenated blood colour (Fig. 1D). Overall, oxygenated blood colour was enhanced more than deoxygenated (t_50_ = 8.753; p<0.001).

In the two-dimensional transform participants decreased deoxygenated blood colour (ΔE = −3.10±0.11, t_50_ = 29.295, p<0.001) and increased oxygenated blood colour (ΔE = 3.03±0.07; t_50_ = 41.473, p<0.001) to optimise healthy appearance. The combined colour change in the two-dimensional transform included an increase in the overall redness and small decrease in overall blueness of the faces (Table S6). These results indicate that participants preferred skin colour consistent with an elevated blood perfusion and blood oxygenation state.

For the cross-cultural study, participants increased the redness of faces to optimise healthy appearance (Δa* = 2.39±0.18 t_49_ = 13.661; p<0.001; Fig. 2B), and redness change applied was negatively related to initial face redness (F_1,1748_ = 187.272; p<0.001; Table S5). Broad agreement between the participants was again found, with all but three participants (92%) showing negative relationships between initial face redness and redness change applied and all but two (95%) increasing redness in the faces (fig. 2C), though there was an interaction between initial face redness and participant ID (F_37,1748_ = 2.211; p<0.001), and an effect of participant ID (F_37,1748_ = 3.256; p<0.001).

For analysis of the effect of face ethnicity on colour manipulations, mixed and south Asian groups were excluded due to small sample size. Face ethnicity (F_2,2.95_ = 0.602; p = 0.604) and participant ethnicity (F_1,21.04_ = 0.133; p = 0.719) did not affect the amount of colour change applied to optimise healthy appearance. An interaction was found between the two (F_2,1551_ = 8.230; p<0.001), with African participants increasing redness more in African faces than in other faces (Fig 2D; Table S3). This indicates a possible own-race effect. In order to optimise healthy appearance, redness was increased in all face ethnicities by both African and UK-based participants.

## Discussion

The healthy appearance of faces is enhanced by increased blood colouration in this study, suggesting that participants interpret skin blood colouration as a cue to underlying health. This is consistent with the established relationship between skin blood perfusion and physiological status. Increased vasodilation and vascularisation of the skin leads to increases in skin blood colour. These processes are enhanced by increased levels of sex hormones in women [35], and by physical training [36]. Skin blood flow is reduced in patients with hypertension [37], type 2 diabetes [38], senescence [39] and in smokers [40].

Blood oxygenation is associated with health and physical fitness [41], whereas deoxygenated blood is associated with ill health [16]. In the current study, the participants preferred oxygenated to deoxygenated blood colour when optimising the healthy appearance of faces. These results show that human observers are sensitive to the subtle colour difference between oxygenated and deoxygenated blood (oxygenated blood is a bright red colour, deoxygenated blood has a slightly bluish red colour; [16]), and interpret this difference in skin blood oxygenation colour as a cue to the health status of individuals. The increased preference for blood oxygenation colour in female faces over male faces may be attributable to the sex difference in cardiovascular performance [42].with for example higher levels of haemoglobin and arterial oxygen content of male blood. This may increase the benefit of oxygenation colour to female faces, and also allow male faces to receive more deoxygenated blood colour with less of a detrimental effect on health appearance. Indeed, it has been suggested that the maximum sensitivities of the medium and long wavelength cones in the retinas of routinely trichromatic primates (a group which includes humans) are ideally suited for identifying small changes in blood perfusion and oxygenation in the skin of conspecifics [43]. The results of the current study suggest that the ability to perceive health cues provided by skin blood perfusion and oxygenation may be an additional advantage of trichromatic colour vision in primates.

Though a possible own-race effect was observed with African participants increasing redness in black faces more than UK-based participants, the effect of facial reddening on apparent health of human faces does not differ according to ethnicity of face or of participant, suggesting that the cues obtained from facial redness are similar in different ethnic groups, and are not affected by culture. Indeed, blushing responses are similar in groups of differing skin darkness, despite the differing visibility of these physiological changes [28], [44].

Several species of birds, fish and primates display colourful ornaments or brightly coloured skin regions, the size and brightness of which reflect aspects of underlying physiology including immune [45], [46], [47], [48], hormonal [3], [4], [6] or reproductive status [3], [8], or social status [6], [7]. As such, coloured ornaments can be considered cues to health. In many cases, these colourful regions influence the behaviour of conspecifics, including agonistic conflicts between males [2], [7], feeding of offspring by parents [1] and mate choice. Larger, brighter ornaments reflect better health status in the bearer and are preferred by, or solicit greater visual attention from, the opposite sex, suggesting that these ornaments act as a cue to mate quality [4], [5], [45], [48], [49], [50], [51], [52].

It has been suggested that skin condition may reliably signal mate value [53], [54], [55], [56] and MHC heterozygosity [57] in humans. Studies have shown that skin colour distribution affects the appearance of health, age and attractiveness in human faces [17], [18], and that skin texture and colour associated with health strongly affects the attractiveness of human faces [58], [59]. In humans, skin vascularisation and vasodilation determine the blood colour of the skin, and are associated with health status. In the current study, we show that colour associated with skin blood perfusion and oxygenation affects the healthy appearance of human faces. Attractiveness, thought to signal underlying health [60], [61], and strongly related to perceived health [60] is a major factor in human mate choice, particularly by men [62]. It is likely, therefore, that the enhanced health appearance associated with increased skin blood colour and oxygenation colour has consequences for attractiveness and mate choice.

## Supporting Information

Table S1 Colour transform applied to produce the high colour endpoint image. The sign is changed for the low colour endpoint image.
(0.02 MB PDF)

Table S2 Colour changes along component CIELab axes and total colour change (ΔE). Mean change across participants and faces (±SE) in the single-axis pigment transforms to maximize health.
(0.02 MB PDF)

Table S3 Effects of participant ID and initial face colour on colour change applied to optimise healthy appearance.
(0.01 MB PDF)

Table S4 Effects of the analysis of face sex and participant sex on amount of colour change applied. An effect of participant ID was found for oxygenated and deoxygenated trials, suggesting that participants behave differently from each other. 70% of participants increase deoxygenated blood colouration and 97% of participants increase oxygenated blood colouration in faces to optimise healthy appearance.
(0.01 MB PDF)

Table S5 Overall CIELab and ΔE colour change in two-dimensional pigment trials.
(0.02 MB PDF)

Table S6 Effects of face ethnicity and participant ethnicity of colour change applied in the cross-cultural study.
(0.01 MB PDF)
