# Supplementary material for: Skin Blood Perfusion and Oxygenation Colour Affect Perceived Human Health
Source: PLoS One. 2009 Apr 1;4(4):e5083. doi: 10.1371/journal.pone.0005083 (PMC2659803; doi:10.1371/journal.pone.0005083)
Supplement: Table S1 — Colour transform applied to produce the high colour endpoint image. The sign is changed for the low colour endpoint image. (0.02 MB PDF) [file pone.0005083.s001.pdf]

	L*	a*	b*	Colour change (ΔE)
**Oxygenated blood colour**	+2.25	+5.36	-0.17	5.81
**Deoxygenated blood colour**	-4.98	+4.29	-1.57	6.75

Table S1. Colour transform applied to produce the high colour endpoint image.

The sign is changed for the low colour endpoint image.
