# Supplementary material for: Skin Blood Perfusion and Oxygenation Colour Affect Perceived Human Health
Source: PLoS One. 2009 Apr 1;4(4):e5083. doi: 10.1371/journal.pone.0005083 (PMC2659803; doi:10.1371/journal.pone.0005083)
Supplement: Table S2 — Colour changes along component CIELab axes and total colour change (ΔE). Mean change across participants and faces (±SE) in the single-axis pigment transforms to maximize health. (0.02 MB PDF) [file pone.0005083.s002.pdf]

Transform	ΔL* component	Δa* component	Δb* component	Total colour change (ΔE)
**Oxygenated blood**	0.57±0.05	1.36±0.12	-0.04±0.00	1.52±0.13
**Deoxygenated blood**	-0.39±0.14	0.34±0.12	-0.12±0.04	0.51±0.19

Table S2. Colour changes along component CIELab axes and total colour change (ΔE).

Mean change across participants and faces (±SE) in the single-axis pigment transforms to maximize health.
