# Supplementary material for: Skin Blood Perfusion and Oxygenation Colour Affect Perceived Human Health
Source: PLoS One. 2009 Apr 1;4(4):e5083. doi: 10.1371/journal.pone.0005083 (PMC2659803; doi:10.1371/journal.pone.0005083)
Supplement: Table S3 — Effects of participant ID and initial face colour on colour change applied to optimise healthy appearance. (0.01 MB PDF) [file pone.0005083.s003.pdf]

	Trial type		
	Deoxygenated	Oxygenated	Cross-Cultural
**Participant ID**	$F_{29,1410}$=1.069; p=0.367	$F_{29,1410}$=1.418; p=0.070	$F_{37,1748}$=3.256; p<0.001
**L***	$F_{1,1410}$=0.110; p=0.740	$F_{1,1410}$=0.148; p=0.700	$F_{1,1748}$=19.527; p<0.001
**a***	$F_{1,1410}$=279.782; p<0.001	$F_{1,1410}$=237.100; p<0.001	$F_{1,1748}$=187.272; p<0.001
**b***	$F_{1,1410}$=34.400; p<0.001	$F_{1,1410}$=2.416; p=0.120	$F_{1,1748}$=0.682; p=0.409
**ID*L***	$F_{29,1410}$=0.895; p=0.627	$F_{29,1410}$=1.352; p=0.101	$F_{37,1748}$=4.387; p<0.001
**ID*a***	$F_{29,1410}$=1.579; p=0.026	$F_{29,1410}$=2.057; p=0.001	$F_{37,1748}$=2.211; p<0.001
**ID*b***	$F_{29,1410}$=0.970; p=0.511	$F_{29,1410}$=0.780; p=0.793	$F_{37,1748}$=1.636; p=0.010

Table S3. Effects of participant ID and initial face colour on colour change applied to optimise healthy appearance.
