# Supplementary material for: Skin Blood Perfusion and Oxygenation Colour Affect Perceived Human Health
Source: PLoS One. 2009 Apr 1;4(4):e5083. doi: 10.1371/journal.pone.0005083 (PMC2659803; doi:10.1371/journal.pone.0005083)
Supplement: Table S4 — Effects of the analysis of face sex and participant sex on amount of colour change applied. An effect of participant ID was found for oxygenated and deoxygenated trials, suggesting that participants behave differently from each other. 70% of participants increase deoxygenated blood colouration and 97% of participants increase oxygenated blood colouration in faces to optimise healthy appearance. (0.01 MB PDF) [file pone.0005083.s004.pdf]

	Trial Type	
	Oxygenated	Deoxygenated
Face Sex	$F_{1,1495}$=4.095; p=0.043	$F_{1,1495}$=4.870; p=0.027
Participant Sex	$F_{1,28.10}$=1.533; p=0.226	$F_{1,28.07}$=1.038; p=0.317
Face Sex*Participant Sex	$F_{1,1495}$=0.028; p=0.867	$F_{1,1495}$=0.092; p=0.762
Participant ID(Participant sex)	$F_{28,1495}$=18.563; p<0.001	$F_{28,1495}$=24.869; p<0.001
L*	$F_{1,1495}$=1.506; p=0.220	$F_{1,1495}$=0.430; p=0.512
a*	$F_{1,1495}$=222.342; p<0.001	$F_{1,1495}$=279.296; p<0.001
b*	$F_{1,1495}$=4.841; p=0.028	$F_{1,1495}$=20.445; p<0.001

Table S4. Model effects of the analysis of face sex and participant sex on amount of colour change applied. An effect of participant ID was found for oxygenated and deoxygenated trials, suggesting that participants behave differently from each other. 70% of participants increase deoxygenated blood colouration and 97% of participants increase oxygenated blood colouration in faces to optimise healthy appearance.
