# Supplementary material for: Skin Blood Perfusion and Oxygenation Colour Affect Perceived Human Health
Source: PLoS One. 2009 Apr 1;4(4):e5083. doi: 10.1371/journal.pone.0005083 (PMC2659803; doi:10.1371/journal.pone.0005083)
Supplement: Table S5 — Overall CIELab and ΔE colour change in two-dimensional pigment trials. (0.02 MB PDF) [file pone.0005083.s005.pdf]

Colour axis	Colour change in 2D blood trials	
	Significance	Mean+SE
**ΔL***	t_{50}=44.659; p<0.001	1.53+0.03
**Δa***	t_{50}=54.343; p<0.001	2.21+0.04
**Δb***	t_{50}=7.356; p<0.001	0.06+0.01
**Overall ΔE**	t_{50}=64.033; p<0.001	2.70+0.04

Table S5. Overall CIELab and ΔE colour change in two-dimensional pigment trials.
