# Supplementary material for: Skin Blood Perfusion and Oxygenation Colour Affect Perceived Human Health
Source: PLoS One. 2009 Apr 1;4(4):e5083. doi: 10.1371/journal.pone.0005083 (PMC2659803; doi:10.1371/journal.pone.0005083)
Supplement: Table S6 — Effects of face ethnicity and participant ethnicity of colour change applied in the cross-cultural study. (0.01 MB PDF) [file pone.0005083.s006.pdf]

Term	Statistics
**Face Ethnicity**	$F_{2,2.95}$=0.602; p=0.604
**Participant Ethnicity**	$F_{1,21.039}$=0.133; p=0.719
**Face Ethnicity*Participant Ethnicity**	$F_{2,1551}$=8.230; p<0.001
**Participant ID(Participant Ethnicity)**	$F_{36,1551}$=28.070; p<0.001
**L***	$F_{1,1551}$=11.856; p=0.001
**a***	$F_{1,1551}$=81.554; p<0.001
**b***	$F_{1,1551}$=1.225; p=0.269

Table S6. Effects of face ethnicity and participant ethnicity of colour change applied in the cross-cultural study.
